# Arginine, scurvy and Cartier's "tree of life"

**DOI:** 10.1186/1746-4269-5-5

**Published:** 2009-02-02

**Authors:** Don J Durzan

**Affiliations:** 1 Department of Plant Sciences, University of California MS 6, One Shields Ave, Old Davis Rd, Davis, CA 95616, USA

## Abstract

Several conifers have been considered as candidates for "Annedda", which was the source for a miraculous cure for scurvy in Jacques Cartier's critically ill crew in 1536. Vitamin C was responsible for the cure of scurvy and was obtained as an Iroquois decoction from the bark and leaves from this "tree of life", now commonly referred to as arborvitae. Based on seasonal and diurnal amino acid analyses of candidate "trees of life", high levels of arginine, proline, and guanidino compounds were also probably present in decoctions prepared in the severe winter.

The semi-essential arginine, proline and all the essential amino acids, would have provided additional nutritional benefits for the rapid recovery from scurvy by vitamin C when food supply was limited. The value of arginine, especially in the recovery of the critically ill sailors, is postulated as a source of nitric oxide, and the arginine-derived guanidino compounds as controlling factors for the activities of different nitric oxide synthases. This review provides further insights into the use of the candidate "trees of life" by indigenous peoples in eastern Canada. It raises hypotheses on the nutritional and synergistic roles of arginine, its metabolites, and other biofactors complementing the role of vitamin C especially in treating Cartier's critically ill sailors.

## Background

One of the first documented uses of indigenous medicine in North America was the cure in the winter of 1536 of Jacques Cartier's crew from a disease he called "Scorbut"(scurvy) [1,2]. Cartier's second voyage (1535–1536) was undertaken at the command of King François 1^er ^to complete the discovery of the western lands under the same climate and parallels as in France. At Stadaconna, now Quebec City, Cartier's crew was cured from scurvy by ascorbic acid (vitamin C) obtained as a decoction from the Iroquois. It was prepared by boiling winter leaves and the bark from an evergreen tree. The tree, identified as "Annedda", became known as the "tree of life" or "arbre de vie" because of its remarkable curative effects. In the winter, scurvy was the most prevalent disease among the Iroquois. This was due to the lack of food and vitamin C [3].

The cure for scurvy was significant for future naval explorations and for the introduction of the tree into France during the Reformation when the Age of Reason began (1558–1648) [4]. The medicinal value of the tree of life contributed to the resurrection of botany, which at that time struggled to free itself from pharmacy when medical men were still its masters. By the eighteenth century, the French naturalists at the Jardin du Roi in Paris knew of *Thuja occidentalis *as the tree of life and planted an avenue of it in the Jardin itself [5].

The Iroquois referred to the tree as Annedda (l'Annedda, Aneda, Anneda, Hanneda) [2]. Other tribal names for conifers were "ohnehta" for white pine, "onita" and "onnetta" for white spruce (Mohawk, Onandaga). These names represent the evergreen nature characteristic of coniferous trees. Regarding the transmission of the tree of life to France, the earlier one goes, the sparser are the available manuscripts. The pre-Linnaeus terminology for conifers made their precise identity impossible to make. Based on collections by French explorers and the ethnomedicine of indigenous peoples in eastern Canada, the true identity of the tree of life became controversial [2]. The identity of Anneda was narrowed down to eastern white cedar or arborvitae *(Thuja occidentalis *L.*)*, white spruce *(Picea glauca *(Moench) Voss), black spruce *(Picea mariana (Mill.))*, eastern white pine (*Pinus strobus *L.), red pine (*Pinus resinosa *Aiton), balsam fir (*Abies balsamea *(L.) Mill.), eastern hemlock (*Tsuga canadensis *(L.)), and juniper (*Juniperus communis *L.*) *[2,6].

We now know that during late a severe winter and at a similar latitude to Quebec City, the candidate trees of life are a rich nutritional source of arginine, proline and other amino acids [7-9]. Their physiological fluids and proteins contain amino acids which are essential in the human diet because the body does not synthesize them (*viz*., phenylalanine, valine, threonine, tryptophan, isoleucine, methionine, leucine, and lysine). Arginine, cysteine, glycine, glutamine, histidine, proline, serine and tyrosine are conditionally essential, meaning they are not normally required in the diet, but must be supplied to specific populations that do not synthesize these amino acids in adequate amounts [10]. Today, these amino acids are used as nutritional support for the recovery of critically ill patients [11-14]. In the recovery from scurvy they would help to promote vitamin C-dependent collagen biosynthesis, promote wound healing, reduce susceptibility to sepsis, and contribute to weight gain [10,15-17].

The importance of arginine as a source of the free radical nitric oxide (NO) was recognized by the Nobel Prize in Physiology or Medicine in 1998 given to R. Furchgott, J. Ignarro and F. Murad. They identified NO as a signalling molecule in the cardiovascular system. Derived from arginine and oxygen, NO maintains blood pressure by dilating blood vessels, helps kill invaders in the immune response, and is needed for wound repair [18-21]. It can pass through biological membranes, oxidize foreign substances, and acts as a secondary biological messenger which affects diverse metabolic pathways. Unexpectedly, the candidate trees of life can produce NO from arginine, and contain guanidino compounds that may regulate the enzyme, nitric oxide synthase (NOS), which is responsible for the formation of NO [8,22,23]. The arginine-derived guanidino compounds are potential therapeutic agents for the regulation of various nitric oxide synthases (NOSs) when the overproduction of NO becomes associated with septic shock, neurodegeneration, and inflammation [21].

My review deals with the nitrogenous compounds in the physiological fluids of candidate trees of life in a severe winter, and theorizes as to how these nutrients could have helped to improve the recovery of the critically ill sailors in 1536. The availability of arginine, the essential amino acids, and biofactors in decoctions, obtained from the trees of life, are discussed in terms of their contributions to the recovery of the critically ill crew at Stadaconna, the ethnnobotany and ethnomedicine of the indigenous peoples in eastern Canada during food shortages, and in the vitamin C-dependent cure for scurvy. This is meant to generate hypotheses, not to confirm them.

### The recovery from scurvy in Jacques Cartier's crew in 1536

Scurvy is an acute chronic illness caused by a dietary deficiency of ascorbic acid (vitamin C). Humans are not able to synthesize vitamin C from glucose because they lack a gluconolactone oxidase [10]. There are two active forms of vitamin C: L-ascorbic acid and dehydroascorbic acid. Ascorbic acid is absorbed by the small intestine and requires an energy-dependent active transport system. It is stored in all tissues. Exposure to long periods of cold temperatures can lead to ascorbic-acid insufficiency.

The first symptoms of scurvy occur when the total-body pool of vitamin C falls below five grams. The body requires vitamin C to efficiently use carbohydrates, fats, and protein. It binds and neutralizes the tissue-damaging effects of free radicals. It is an essential cofactor for the formation of collagen, the body's major building protein, and is essential to the proper functioning of all internal organs.

Scurvy is characterized principally by anemia, hemorrhagic manifestations in the skin (ecchymoses and perifollicular haemorrhage), and in the musculoskeletal system (haemorrhage into periosteum and muscles). The gums start to bleed. Teeth are loosened [24]. With no vitamin C intake, the symptoms of scurvy would occur after one to three months. Unless treated, scurvy is fatal.

At Stadaconna (46° 49' N, 71° 13' N) and in November1535, Canada's cold struck with its entire rigor, and ice thickened to two fathoms. In December, over 50 of the Iroquois died from an unknown sickness (scurvy). The sickness began to spread to Cartier's crews in all three of his ships. By mid-February 1536, of the 110 member crews, 8 were already dead and more than 50 past all hope of recovery. Excerpts from Burrage [1, p. 73] reveal that the unknown sickness in Cartier's crew "*spread itselfe amongst us after the strangest sort that ever was eyther heard of or seene, insomuch as some did lose all their strength, and could not stand on their feete, then did their legges swel, their sinnowes shrinke as blacke as any cole. Others also had all their skins spotted with spots of blood of a purple colour: then did it ascend up to their ankels, knees, thighes, shoulders, armes and necke: their mouth became stincking, their gummes so rotten, that all the flesh did fall off, even to the rootes of teeth, which did also almost fall out*".

*"Our Captaine seeing this our misery, and that the sicknesse was gone to farre, ordained and commanded, that every one should devoutly prepare himselfe to prayer, and in remembrance of Christ, caused his Image to be set upon a tree, about a flight shot from the fort amidst the yce and snow, giving all men to understand, that on the Sunday following, service should be said there, and that whosoever could goe, sicke or whole, should go thither in Procession, singing the seven Psalmes of David, with other Letanies, praying most heartily that it would please the said our Christ to have compassion upon us ... That day Philip Rougemont...being 22 yeeres olde, and because the sicknesse was to us unknown, our Captaine caused him to be ripped to see if by any meanes possible we might know what it was...he was found to have his heart white, but rotten, and more than a quart of red water about it: his liver was indifferent faire, but his lungs blacke and mortified, his blood was altogither shrunke about the heart, so that when he was opened great quantitie of rotten blood issued out from about his heart...Moreover, because one of his thighs was very blacke without, it was opened, but within it was whole and sound." *Scurvy continued to spread until not more than three sound men remained in the ships. None were able to go under the hatches to "*draw drink for himselfe, nor for his fellowes*."

At Stadaconna, Cartier encountered the native Domagaia, who *"not passing ten or twelve dayes afore, had bene very sike with that disease, and had his knees swolne as bigge as a childe of two yeres old, all his sinews shrunke together, his teeth spoyled, his gummes rotten, and stinking. Our Captaine seeing him whole and sound, was therat marvelous glad, hoping to understand and know of him how he had healed himselfe...He answered, that he had taken the juice and sappe of the leaves of a certain Tree, and therewith had healed himselfe: For it is a singular remedy against that disease."*

Domagaia "*sent two women to fetch some of it, which brought ten or twelve branches of it, and therewithall shewed the way how to use it... to take the barke and leaves of the sayd tree, and boile them togither, then to drinke of the sayd decoction every other day, and to put the dregs of it upon his legs that is sicke: moreover, they told us, that the vertue of that tree was, to heale any other disease: the tree in their language called Ameda or Hanneda...*" Other translations refer to the tree as "*Annedda*", "*Anneda*" or "*Hanneda*" [2]. This sickness was treated with a boiled decoction from the bark and leaves of "*a tree as big as any oak in France*".

Cook [25] translates that "*The Captain at once ordered a drink to be prepared for the sick men but none of them would taste it. At length one or two thought they would risk a trial. As soon as they had drunk it they felt better, which must clearly be ascribed to miraculous causes; for after drinking it two or three times they recovered health and strength and were cured of all the diseases they had ever had. And some of the sailors who had been suffering for five or six years from the French pox [syphilis] were by this medicine cured completely. When this became known, there was such a press for the medicine that they almost killed each other to have it first; so that in less than eight days a whole tree as large and as tall as any I ever saw was used up, and produced such a result that had all the doctors of Louvain and Montpellier been there, with all the drugs of Alexandria, they could not have done so much in a year as did this tree in eight days; for it benefitted us so much that all who were willing to use it recovered health and strength, thanks be to God*." We do not know how much ascorbic acid was lost during the boiling of the decoction and in the recovery of the "dregs", but it is clear that sufficient vitamin C was available to initiate a cure.

In today's healthy men, the body is estimated to store 1,500 mg of ascorbic acid. It is used at an average rate of 3% of the existing pool per day [26]. After three months of vitamin C deprivation, the stores become largely depleted. The earliest signs of depletion begin during the first month of deprivation. Bleeding gums are not the most characteristic feature of scurvy and are a late manifestation. Except in the most severe cases, vitamin C would stop spontaneous bleeding within 24 hours and bleeding of the gums would stop in two to three days. Muscle and bone pain would quickly fade [24].

In advanced scurvy another group of symptoms becomes identifiable [15,24]. They include ocular haemorrhages, loss of secretion of salivary and lachrymal glands, swelling of the parotid and submaxillary glands, loss of hair, femoral neuropathy, oliguria with edema of the lower extremities, psychological disturbances, impaired vascular activity, poor responses to stimuli that normally activate vasomotor adaptive mechanisms, and scorbutic arthritis, which is clinically similar to rheumatoid arthritis with pain, swelling, joint effusions, and limited motion. All of the above would respond completely to therapy with ascorbic acid given the added nutritional benefits of the conditionally and essential amino acids and other biofactors in the decoction.

### Identities of Annedda and the trees of life

Before 1547 and during the reign of François 1^er^, seeds of Annedda were delivered to the Royal Garden (Jardin du Roi) at Fontainbleau and presented to the King. Apparently seeds were collected from a tree or trees similar to Annedda [2]. In 1553, Belon wrote in the Bulletin Dendrologique that Annedda was growing in the Royal Gardens at Fontainbleau. Nearby was another small tree, a five-needled pine, referred to as the second tree of life. Wood from these trees were used as medicine.

In Hickel's translation [27] of Belon's book, we read that "*...à cette époque, les seules espèces exotiques introduites étaient l'Arborvitae (Thuya occidentalis) et Pinus strobus, et que, d'autre part l'auteur confond plus ou moins diverses espèces de pins*." When Belon visited Turkey, he found a tree similar to the one at Fontainebleau, which was brought from Canada and called "Arbre de Vie". Moore [6] citing the works of Bolle [28] and Annon. [29], who both reexamined Belon's records, proposed that the identity of the Annedda was not eastern white cedar, but a five-needled white pine (*Pinus strobus)*. It is now evident that two trees of life were introduced from North America as exotic species [2,6]. "*The fate of the pine at Fontainbleau is not known" *[6]. Bolle [28]*"could not find any further record of eastern white pine growing in Europe until 150 years later when it was introduced into England*".

In 1632, the botanical garden, established in Paris in 1632 by King Louis XIII of France, was intended for the cultivation of medicinal plants [5]. Landowners and naturalists were engaged in testing the effects of climate upon growing new exotic species arriving in France. A Bridgeman Art Library archive shows a "burgeoning bower" resembling eastern white pine in the botanical garden (Nature 2001, **410**, 303). The King's garden survived the French Revolution (1796–1798) and its nurseries were used to provide patriotic 'trees of liberty'. They were planted in front of public buildings. The first trees of liberty were actually maypoles planted by peasants as a symbol of revolt against local lords in the winter of 1790.

Today, Annedda is commonly referred to as eastern white cedar or arborvitae *(Thuja occidentalis *L.*)*. This appellation was based on botanical evaluations, historical documents, naval and folklore medicine, notes of Cartier's contemporaries, and on the estimates of biochemical content of vitamin C [2]. The anti-scorbutic benefits of the candidate trees of life are abundant in the records and reviews of indigenous Maritime medicine [2,3,30-33].

Conifers, native along the travel routes of Jacques Cartier, and with known high levels of vitamin C are *Picea rubens*, *Pinus resinosa*, *Pinus nigra*, and *Pinus banksiana*. In the "Native Trees of Canada", Canada Forest Service Bulletin 1919, No. 61 the botanical names of conifers had popular names. *Thuja occidentalis *was called cedar, and referred to as white cedar, and arborvitae. *Pinus strobus *was called white pine, and sometimes referred to as Weymouth pine, pattern pine, eastern white, yellow, and Quebec pine. *Picea canadensis *was called white spruce, and sometimes northern, skunk, cat spruce, and pine. *Pinus banksiana *was called jack pine and sometimes grey pine, cypress, juniper, and Banksian pine.

Today, the eastern white cedar (*Thuja occidentalis *L.) has the largest number of cultivars, and many do not resemble the species type [34]. Mature trees will reach 30 to 40 feet tall with a spread of 15 feet. The upright cultivars are much shorter. The latter would unlikely be "*as big as any oak in France*". In eastern Canada, white pine was reported to reach a height of 250 feet and a diameter of 6 to 15 feet [35].

### Conifer decoctions for the treatment of scurvy

Domagaia cured himself with the "*the juice and sappe of the leaves of a certain tree*". Adult scurvy is now treated with 300–1000 mg of ascorbic acid per day [15]. In clinical dermatology, ascorbic acid is recommended three times a day, 100 mg is given until 4 g is reached, and then 100 mg/d becomes curative in days to weeks [24]. Repletion studies demonstrated recovery from daily doses of only 6.5 [26]. Larger doses gave more rapid improvement and increased ascorbic acid storage in the body. The plasma levels of ascorbic acid attained depended on body weight (dose per kg of body weight) and on whether or not any prior depletion had been adequately repleted [16].

In early explorers, a deficiency of vitamin C repeatedly caused morbidity and death [36,37]. Teas, brews, and beers, prepared from the needles of spruces and pines, were used to treat the symptoms of scurvy [38,39]. Scurvy remedies were being made, sold and used under the name of "sapinette"[2].*"According to the physician Gardane, in Des maladies des créoles (Paris 1784), this was a decoction of "sapin du Nord", or Picea abies. In Canada sapinette was made from the buds of the "Prussian fir", a name which was used indiscriminately for Abies alba, A. balsamea, and Picea abiesby Cartier. Sapinette was widely used in Canada, but the recipe seems to have come originally from the Baltic coast and sapinette was being used by the Russian navy long before the French took interest in it. The Russians in fact did use fermented pine buds with their fir decoction, though the species here is not specified. But it seems the French used fir, even in Canada" *(Spary, personal communication [5]).

Spary writes, "*The French were experimenting with sapinette on their long-distance voyages during the 1780s, and it was stocked on board the Laperouse expedition vessels"..." sapinette was bought ready-made from London. All things considered, this does not suggest that there was a direct connection between Pinus strobusin particular and the antiscorbutic programme, though it is entirely possible that this species was brought to Paris to be investigated for its virtues in that regard*". The British knew of the anti-scorbutic benefits of sapinette and of lemons and oranges in a cure for scurvy [37]. In 1753 scurvy was recognized by the British medical community as directly related to dietary deficiency.

Spruce beer was used as an anti-scorbuticum by James Cook in his second Pacific voyages in Western Canada (1772–1775) [40]. Cook obtained this recipe for spruce beer from Joseph Banks who had visited Newfoundland before Cook [41]. The beer was prepared from fresh needles of a spruce tree, which in New Zealand was *Dacrydium cupressinum *[42]. On Cook's third voyage near Alaska, Sitka spruce (*Picea sitchensis*) was used but it was not as acceptable as the brew from *Dacrydium*. [43]. A similar drink called "Kallebogas" was used in Newfoundland. Variations involved the addition of rum and maple sugar [44].

Vitamin C was first isolated from paprika, chemically identified, and its metabolic role worked out by Albert Szent-Györgyi. He found that vitamin C also required cofactors to function properly. These cofactors are now known to be flavonoids. He was awarded the Nobel Prize in Physiology or Medicine in 1937 for his discoveries in biological combustion with special reference to vitamin C and for the catalysis of fumaric acid, an intermediate in the citric acid cycle. These factors, taken together, were probably available in the decoction used to cure scurvy.

Eastern hemlock (*Tsuga canadensis*) and black spruce (*Picea mariana*) served as ascorbutica [32,44]. The indigenous peoples of the Maritime Provinces of Canada used roots, twigs, leaves, and bark, but rarely strobili or seeds in decoctions taken as a cupful in the morning [3]. Teas, prepared by steeping or boiling leaves from conifers, served as refreshing drinks and a tonic of medicinal value [33]. Green tissues offer high moisture content, vitamin C, folic acid, minerals and other biofactors. Roots are a good source of minerals but provide only small amounts of vitamins in a 100-gram portion [32]. The bark was usually collected from the east side of the tree. The selected root or branch ran to the east [3]. The reason was that these collections benefited from having more potency obtained from sunlight.

The vitamin C in lemon and oranges (50 mg/100 g) are exceeded in the needles and bark of several conifer spp. [33]. Reduced ascorbic acid in 100 g of fresh needles and shoots was reported in *Abies balsamea *(270 mg), *Picea rubens *(169 mg), *Pinus strobus *(32 mg, bark contained 200 mg), *Thuja occidentalis *(45 mg) [2]. R. B. Thomson at the University of Toronto found a content of 20–80 mg reduced ascorbate in 100 g of white spruce bark [2]. For the treatment of scurvy, spruce (white and black) was considered as a likely candidate for the tree of life based on the ethnobotanical literature. Spruce is frequently recorded as being antiscorbutric and common in Quebec City. White pine was also widely used.

The extracts from Cartier's tree of life raised considerable interest as a cure for all diseases. In 1494 King Charles VIII of France had already invaded Italy. Within months, his army collapsed and was routed not by the Italian army but by a mysterious new disease [45]. The disease was spread through sex and killed many of Charles's solders. European physicians were already aware of the root of sarsaparilla (*Smilax officinalis*) as a tonic, blood purifier, diuretic, and sweat promoter. Cartier's claim for the Iroquois decoction as a cure of all diseases may have been overstated to impress King François 1^er ^(1515–1547). It is unlikely that vitamin C and other components from the trees of life would have cured syphilis in Cartier's crew at Stadaconna.

### Food sources and medicines used by indigenous peoples in Canada

Twenty-five conifer species are identified as a traditional food source by indigenous peoples of Canada. Kuhnlein and Turner [32] have described the distribution, occurrence, food values and warnings for the Cypress family (Cupressacae), the Pine family (Pinaceae), and the Yew family (Taxaceae). The Canadian indigenous peoples are recognized for their ingenuity in processing foods to remove toxins by heating, leaching, fermenting, adsorption, drying, physical processing, and changing the acid-base ratio. Arnason et al. [33] have produced a compendium of the various uses of plants, which would have been accessible to Cartier before winter, and used by indigenous peoples of eastern Canada.

Land-based scurvy is well documented as occurring during times of food shortages [46]. Late springs and low levels of vitamin C in stored grain contributed to endemics of sub-clinical scurvy. As for the candidate trees of life, food would include seeds, buds, inner bark, cambium and sap of trees. The inner bark of trees was eaten at any time of the year as an emergency food. The inner bark of hemlock was not an adequate famine food on its own if harvested outside the spring season [33].

Needles of *Abies*, *Picea*, and *Pinus *spp. have protein content from 2.5 to 8.8 mg/100 g fresh weight [32]. Arginine, a dominant amino acid in protein and the physiological fluids of conifers, was first isolated in Switzerland by Schulze [47] using seedlings of *Picea*, *Pinus*, and *Abies spp*.. In seeds, arginine can represent 10% of the N in conifer protein [48]. The sugar, amino acids, and protein content of *Pinus banksiana *seeds from different geographic sources across Canada correlates significantly with climate variables at the seed source [49]. Expanding spruce buds and germinating jack pine seedlings would provide a rich source of amino acids, proteins and nucleic acids [50-56].

Given the statement that "*in less than eight days a whole tree as large and as tall as any I ever saw was used up*", lichens, commonly found on tree trunks and branches, and probably added many biofactors to the nutrients and vitamins in the Iroquois decoction. The main edible tree lichen is *Bryoria fremontii *[32]. Lichens comprise two types of plants, an alga and a fungus growing together in a symbiotic relationship. Lichens are difficult to digest because of their complex polysaccharides, which do not break down during cooking. Some lichens were boiled for 24 h and eaten only in times of food scarcity and to remove toxic substances [32].

In general, tree bark only contains traces (0.04 to 0.17%) of nitrogenous extractives [57,58]. The inner bark of loblolly pine, felled in December at Gulfport, Mississippi in a much warmer climate than Quebec City, was dominated by the amides (glutamine and asparagine), their dicarboxylic acids, and alanine [59]. As for compounds not containing nitrogen in conifer bark, the bioflavonoids and proanthocyanidins are constituents that have been commonly found in medicines. The Algonquin in Quebec used decoctions from *Pinus strobus *to treat breathing disorders, rheumatism, and kidney disorders [33]. *Picea *spp. were used by the Iroquois to treat respiratory ailments, urinary problems as a poultice for blood poisoning [33].

The boiled decoction that cured scurvy was prepared from the bark and leaves of ten or twelve branches. The dregs, which were put on legs, would contain many factors as a salve. In a 20 kg bulk extract from white spruce, the boiled extractives contained at least nine compounds which were weakly reactive with the Sakaguchi reagent for the guanidino group (unpublished data). A total ion chromatogram, mass spectral data, the molecular weight of species in the extract, and a list of their putative elemental composition, revealed several yet unidentified N-containing compounds and polymers having very high molecular weights, one of which had 220 Da subunits.

During a sabbatical at the University of Quebec, Dr. J. Masquelier read Jacques Cartier's account and this turned his attention to the antioxidant proanthocyanidins of conifer bark [60]. He referred to proanthocyanidins as pycnogenols. Pycnogenol^® ^is now a patented trade name for a water extract of the bark of the maritime pine (*Pinus pinaster*) commonly grown in the coastal southwest of France. The trade name refers to a specific proprietary pine bark extract with proanthrocyanidins (procyanidins) from *Pinus maritima*. The procyanidins scavenge free radicals and modulate NO metabolism [61]. They are capable of preventing the oxidation of vitamin C and are more effective than vitamin E in scavenging damaging free radicals. Considerable literature now exists on the health benefits of the procyanidins when taken as supplements. As of 2008, the National Institutes of Health in the USA were carrying out clinical trials of pycnogenol for the treatment of lymphedema, endothelial function in coronary heart disease, hypertension and diabetes.

Herbal remedies from eastern Canadian conifers contain salicylates, astringent tannins, polyacetylenes, antibacterial alkaloids, anti-inflammatory terpenes [33,62,63]. Recognizing *Thuja occidentalis *as the "tree of life", Felter [64] described its medicinal uses in American therapy. He recommended extractives in the form of aqueous, non-alcoholic, ointment and oil preparations.

Indigenous peoples have employed *Taxus *spp. for their utility, wood quality, mythology and medicines [65]. Decoctions were used to treat fever, scurvy, and to bring out clots and alleviate pain after childbirth.*Taxus *spp. contain more than 300 taxanes (taxoids) some of which are poisons, and others have significant physiological effects [66]. Today, the most well known taxane is paclitaxel (Taxol^®^). It blocks cellular growth by binding to microtubules. This property makes it useful for the treatment of various cancers, but with side effects. Taxane-like compounds were detected in spruce and other conifers using antibodies to the taxane ring [23,67,68]. The recovery of paclitaxel and the taxanes from *Taxus *biomass was increased by mechanical stresses which elicit NO production from arginine [22,23]. Product recovery was reduced by adding a synthetic guanidino inhibitor of NOS. We now return to arginine and other amino acids, which are important in human nutrition and serve as substrates required for the vitamin C-dependent synthesis of collagen in the cure for scurvy.

### Arginine and amino acids in overwintering conifers

The seasonal changes in the amino acid composition of conifers represent a tropistic adaptation to environmental changes which prepare trees for dormancy and survival over long and severe winters. Winter decoctions, used by the Iroquois, would have contained the conditionally essential arginine and the essential amino acids required in the human diet. The physiological fluids of *Picea glauca *buds and shoots during a severe winter at Petawawa (45° 08' N, 81° 27' W) and similar in latitude to Quebec City were found to contain between150 to 200 mg amino acid N per 100 g fresh weight [8].

When the first snow appeared in November, the percent contribution of arginine N to the total soluble amino acid N increased from 20 to 45%. It was 40–53% in December. In January and February the shoots became ice-covered and the temperatures averaged -16 C. Arginine N now comprised 30–43% and 12–18% of the total soluble amino acid N, respectively. Levels of glutamine N always remained low, but increased significantly when buds expanded in spring. Proline N, which is derived from arginine, varied between 3 to 5% (November), 4 to 6% (December), 4 to 7% (January), and 8–13% (February).

From November to February, the concentrations in mg of the nine amino acids, essential in human nutrition, and recovered from the physiological fluids of 100 g fresh needles from shoots at 4 to 5 feet were: phenylalanine (0.5–1.5), leucine (0.4–1.6), isoleucine (0.4–1.0), valine (0.5–1.5), lysine (0.7–1.5), threonine (0.9–1.3), methionine, tryptophan, and histidine (each 0.01). Arginine, ranged between 16.0–21.0 mg/100 g fresh weight. The seasonal trends in amino acid content were reaffirmed with excised dormant buds forced to sprout under aseptic conditions in the laboratory [69]. Tissue and bark compositions can vary due to differences in age of tissues in trees, environmental stresses, time of day, and the availability of N from the environment [7,52,53,69-71]. With white spruce saplings, when ammonium is the sole source of N in sand cultures, arginine and guanidino compounds are major components of the total soluble N but not when nitrate is the sole source of N [72,73].

Isotopic tracers are phenotyping tools in metabolism and a cornerstone in nutritional science. In white spruce, [UL-^14^C]-L-arginine serves as a precursor for proline via ornithine [8,9]. Feeding [UL-C^14^]-L-proline and [UL-^14^C]-L-glutamine to winter dormant buds revealed traces of ^14^C-glutamic-γ-semialdehyde and ^14^C-Δ^1^-pyrroline-5-carboxylic acid [73]. These intermediates are highly transient precursors for the synthesis of proline, glutamic acid and glutamine. Free hydroxyproline is not found in the physiological fluids unless the tissues are pathological. ^14^C-Proline and ^14^C-hydroxyproline residues were recovered from protein hydrolysate indicating that proline was hydroxylated to hydroxyproline in spruce protein. L-Proline-4-^3^H incorporation into protein was also observed with seedlings of *Pinus banksiana *[54]. In humans, proline and hydroxyproline obtained from conifer buds and seedlings would be available for the vitamin C-dependent synthesis of collagen.

**Figure 1:**
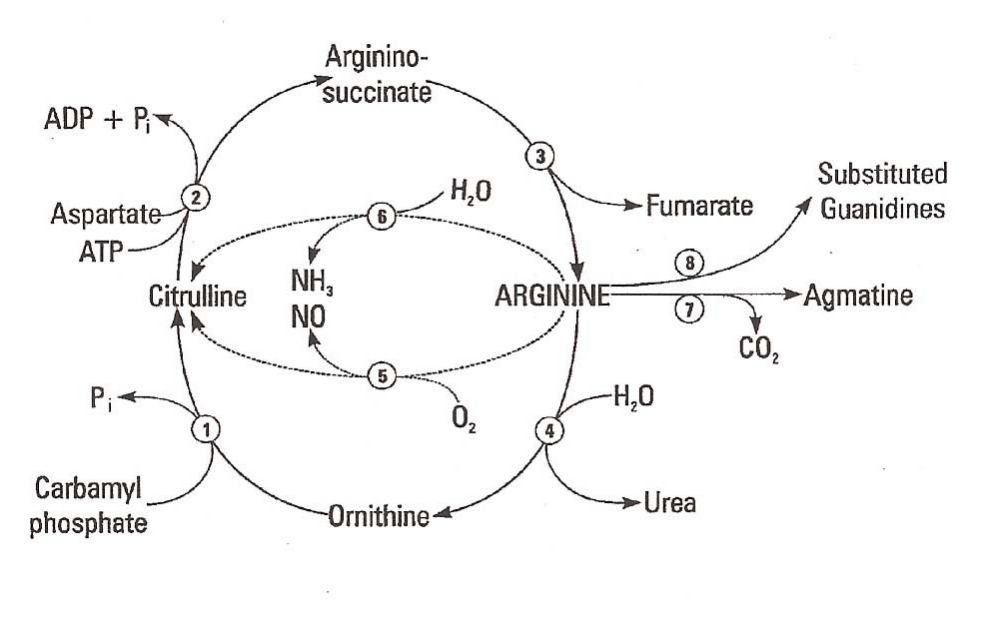
**Arginine metabolism, the partial reactions of the urea cycle, the L-arginine-NO pathway, a citrulline-NO cycle, and a branch point leading to the formation of guanidino compounds with special reference to conifers in eastern Canada**. Enzymes: **1. **ornithine carbamoyl transferase, **2. **argininosuccinate synthetase, **3. **argininosuccinate lyase,**4. **arginase, **5. **nitric oxide synthase, **6. **arginine deiminase, **7. **arginine decarboxylase, **8. **numerous enzymes acting on arginine and responsible for the formation of guanidino compounds. Reactions **1 to 4 **comprise the partial reactions of the urea cycle in plants. Not shown is the synthesis and turnover of proteins which alters the pool of available arginine and other protein amino acids. Reactions **2, 3, and 5 **comprise the L-arginine-NO pathway and the citrulline-NO cycle, which are responsible for the stress-induced formation of NO from arginine and oxygen. **6**. Arginine deiminase is not yet known in conifers but has been reported in other plants. Steps **7 **and **8 **remove arginine from the urea and citrulline-NO cycles and divert N into the naturally occurring guanidino compounds some of which are inhibitors of respiration. During the onset of winter dormancy, arginine N, the guanidino compounds and proline N accumulate in the physiological fluids. Arginine is stored in reserve proteins to provide N for amino acid, amide, and renewed protein and nucleic acid synthesis in the spring. The conversion of proline to glutamine via glutamic acid now provides transferable hydrogen atoms making proline a readily available and highly-water soluble source of energy and reducing power for the photosynthetic assimilation of carbon dioxide.

The metabolic pathways, cycles, and enzymes responsible for arginine metabolism in conifers are summarized in Figure 1. [UL-^14^C]-L-arginine and [carbamoyl-^14^C]-L-citrulline fed to pines and spruces revealed that arginine was synthesized via the intermediate steps of the urea (ornithine) cycle [74-80]. Arginine serves as a pivotal departure point from the urea cycle for the synthesis of urea and ornithine via arginase, and for proline, glutamate and glutamine. It is a substrate for the formation of NO and citrulline via NOS, and for the guanidino compounds and in protein synthesis. Arginase has been purified from conifers [81].

During rapid growth, the fates of ^14^C-urea and tritiated water in white spruce and jack pine tissues were a function of light availability [78,79]. In light and darkness, urea was degraded by urease to ammonia and carbon dioxide. In light, the ^14^C-carbon dioxide, derived from urea, was reassimilated by photosynthesis and incorporated initially into alanine and glutamic acid. In darkness, ammonia and ^14^C-carbon dioxide contributed to the formation of carbamoyl phosphate for the biosynthesis of arginine and other metabolites [80]. In winter dormant tissues, these metabolic steps were latent and difficult to detect. Most of the N in the physiological fluids was stored in arginine and the guanidino compounds.

Amino acids are water soluble at high temperatures [82] and most would be stable in boiled decoctions. Depending on pH, boiling may reduce the levels of glutamic acid due to its conversion to pyroglutamic acid. Vitamin C is readily available in fresh tissues even in winter. Its concentration usually increases during the spring growing season [33].

**Figure 2:**
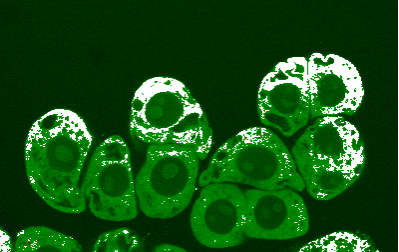
**The production and diffusion of nitric oxide (NO) (white) in the cytoplasm (green) of clusters of conifer cells one hour after mechanical agitation**. NO diffuses into the culture medium around cell clusters. The large dark green circle inside each cell is the nucleus. Some nuclei may contain a nucleolus (light green). The small and irregular dark areas outside the nucleus and in the cytoplasm represent the vacuoles (laser confocal photomicrograph ×400). NO was visualized with a fluorescent probe DAF-2 DA (diaminofluorescein diacetate). NO is derived from L-arginine and oxygen by nitric oxide synthase (NOS) activity in conifers and humans. NOS activity and the production of NO in the conifer cells is blocked by *N*^*ω*^-monomethyl-L-arginine.

The food processing industry has demonstrated that the largest losses of vitamin C in foods occur during heating [17]. We do not know if the "*juice and sappe of the leaves*" from Annedda were obtained without boiling and taken by Domagaia. Fresh conifer leaves can be chewed right off a branch provided that scurvy would prevent him from doing this. Chewing or crushing the leaves would release NO from the conifer cells (Figure 2). The decoction from Annedda, took every other day, provided the needed warmth as a protection from cold, sufficient vitamin C, various biofactors, the essential amino acids, and the conditionally essential arginine, which would provide N for the synthesis of NO in the wasting sailors.

### Arginine as a source of nitric oxide in conifers

The first report of NO production from arginine in a conifer was provided by Magalhaes et al. [83]. Using a dye (Figure 2), bursts of NO were visually detected when cells were wounded or exposed to a variety of and biotic and abiotic stresses [22,23]. NO is synthesized by NOS in the L-arginine-NO pathway. The pathway comprises a citrulline/NO cycle (Figure 1). A citric acid cycle, not shown in Figure 1, provides aspartate N via fumarate, malate, and oxaloacetate for the synthesis of L-argininosuccinate from citrulline.

Plants and animals synthesize NO and citrulline from arginine and oxygen as substrates [22,23,84,85]. The amidine N of the guanidino moiety of arginine is the source of the N in NO. Citrulline is recycled back to arginine via argininosuccinate. Plant NOS remains incompletely characterized and is a different enzyme from the NOSs in humans [84,85]. Atypical of most conifers, the shoots of seedlings of *Cryptomeria japonica *have high levels of citrulline rather than arginine [86].

In conifers, low and protective levels of NO are rapidly produced in response to mechanical forces, environmental stresses, wounding, and as a protective measure against insects and diseases [22,23,85,87-90]. NO activates second messengers which participate in protein phosphorylation, nitrosylation, gene expression, development, and in expressions of adaptive plasticity. NO contributes to developmentally programmed cell death (apoptosis) and autophagy. This is demonstrated in the death of one of the four megaspore cells after meiosis, and again in monozygotic cleavage polyembryony of early embryos found in developing seeds [91,92]. High levels of NO damage DNA and lead to the nitration of the tyrosine residues in cell regulatory proteins [88,89]. NO bursts are produced before the release of ethylene, a plant hormone which is associated with senescence and fruit ripening [93].

NO production from arginine represents a significant evolutionary advance in gymnosperms before angiosperms and humans evolved. Recognition of the natural and synthetic guanidino compounds as inhibitors of NOS was facilitated by the availability of authentic standards, isotopic tracers, and by the development of new analytical instrumentation [94-96].

### Guanidino compounds derived from arginine in conifers

An important physiological property of the guanidino group is its ability to form a bond of high energy with phosphate. This phosphate can be transferred, without the loss of bond energy, to couple and provide energy for biochemical and mechanical processes [10]. Guanidines with the high energy bond are called phosphagens, e.g., *N*-phosphorylarginine. Phosphagens are vital for the sustained action of muscles in many invertebrates and some vertebrates. *N*-phosphorylarginine was reported in jack pine cell cultures [97,98] and in developmental stages in the eastern spruce budworm which feeds on conifer needles [99,100].

In humans, a guanidino-amino acid, creatine is the precursor for phosphocreatine, which serves as the phosphagen and energy buffer [10]. Creatine is synthesized from glycine and receives its guanidino group from arginine. I could find no evidence in spruce buds for the synthesis of creatine from ^14^C-glycine and [1-^14^C]-guanidinoacetic acid. Guanidinoacetic acid inhibited oxygen uptake and yielded ^14^CO_2_, ^14^C-glycine and ^14^C-serine. The transamidination of [1-^14^C]-guanidinoacetic acid with glycine yielded guanidinoacetic acid and ^14^C-glycine. This transamidination occurred as arginine-rich storage proteins in spruce buds were being turned over with a respiratory quotient less than one.

In spruce and pine, γ-guanidinobutyric acid is commonly reported as a product of arginine metabolism [8,9,42,101,102]. The detection of α-keto-δ-guanidinovaleric acid in white spruce suggested that the transamination of arginine with a keto acid followed by decarboxylation would account for the formation of γ-guanidinobutyric acid [103]. In general, the naturally occurring guanidino compounds are derived by decarboxylation, transamidination, transamination, condensation, methylation, phosphorylation, cyclization, and by dehydrogenase activity [104,105].

In the Petawawa forest and after four years of growth in continuous full, 45, 25, and 13% natural light, shade-tolerant white spruce saplings redistributed their biomass before the onset of winter dormancy [71]. A spruce sapling requires at least 20% light to survive. Increased shading diverted soluble N from glutamate, glutamine, and aspartic acid to arginine N mainly in roots and to a lesser extent in stems, buds, and needles. γ-Guandinobutyric acid and several unidentified guanidino compounds accumulated mostly in roots of saplings at 25 and 13% light [unpublished data]. Arginine and the guanidino compounds comprise a N-rich metabolic pathway that helps to account for the ability of conifers to adapt and survive under limiting environmental conditions. Their importance in the food and medicinal practices and as markers of the inappropriate release of NO of the indigenous peoples, requires further investigation. Their role as regulators of NOS would depend on being closely related to the structure of arginine and their ability to bind to NOS.

### Nutritional support in treating scurvy and critical illness

The purpose of nutritional support is to save life, preserve and improve cellular function, and to speed recovery. Critical illness is generally characterized by a combination of starvation and stress. Critical illness can be viewed in four stages [12]: 1. Acute critical illness in response to a stressor. 2. Prolonged acute critical illness. 3. Chronic critical illness. 4. Recovery.

In the first stage, substrates are shunted away from anabolism and toward vital organ support and inflammatory proteins. Nutritional support at this stage remains unproven and may ultimately become detrimental. At stage 2, nutrition and metabolic support to prevent and treat multiple organ dysfunction syndromes become very important. This must be maintained to prevent the deficiency of water-soluble vitamins. Care must be taken not to do harm by inappropriate overfeeding. At Stadaconna, the prescribed remedy of taking the Iroquois decoction "every other day" was consistent with this purpose.

The best-understood function of vitamin C is in the synthesis of collagen [10]. Ascorbate is required for the hydroxylation of proline residues in procollagen. Procollagen is secreted by fibroblasts as a complex of three cross-linked polypeptide chains. Cross-linking requires the formation of covalent bonds between lysine residues. The hydroxylation of proline to hydroxyproline stabilizes the triple helix structure of collagen. This contributes to the formation of fibers, which can take several months, followed by scar formation, wound contraction and healing [106].

Collagen subunits contain a prolyl 4-hydroxylase with one atom of non-heme iron [10]. This enzyme requires α-ketoglutarate and oxygen as substrates. The α-ketoglutarate is oxidatively decarboxylated to carbon dioxide and succinate. A remaining oxygen atom is used to hydroxylate the proline residue in collagen. Heme iron is now oxidized to inactivate the enzyme.

Vitamin C deficiency leads to decreased collagen secretion due to capillary fragility. This is associated with defective connective tissues, poor wound healing, and susceptibility to sepsis.

The synthesis of NO by a heme-containing NOS would increase the circulation of oxygen supply in blood. The lack of vitamin C reduces the hydroxylation reactions needed for the synthesis of carnitine from lysine, and for the hydroxylation of dopamine to norepinephrine. The oxidation of tetrahydrofolic acid, which maintains adequate levels of folic acid and keeps iron in its reduced state, would be prevented [10].

Vitamin C has nutritional value as a co-antioxidant by interacting with vitamin E [107]. In the prevention of disease, intervention studies with vitamin C as an antioxidant have shown no clinical benefit [108]. Arginine supplementation and the availability of other amino acids, such as proline and glutamate, would spare the energy costs in ATP equivalents for their synthesis as substrates for collagen, and other proteins in the body [109].

In the winter, the Iroquois hunted a '*number of wild animals such as fawns, stags, and bears, hares, martens, foxes, otters, and others*" but they were stingy and brought Cartier's crew very few. The ice was extremely thick and the sailors were too weak to fish. They would not be seriously deficient in arginine as long as some meat and fish were available. Proteins are graded by "quality" on the basis of their content of essential amino acids [110], and arginine is conditionally essential. Intakes of 1 to 1.5 g protein (0.16 to 0.24 g N)/dg per d are common practice and usually advised.

In healthy adults, arginine is derived from the diet, endogenous synthesis, and turnover of body proteins. Arginine is usually ingested in diets at a rate of 3 to 5 g/d [111]. A common supplemental dose for arginine is 3 g which is taken by mouth three times a day [13,14]

A kilogram of fresh spruce needles would contain 120 to 160 mg of free arginine N. More arginine would be available if the arginine-rich storage proteins of conifers were digested. Protein, ingested in amounts exceeding those needed to replace body losses, is deaminated. Its nitrogen is excreted as urea in urine [110].

In cases of catabolic stress or conditions involving a dysfunction of the kidneys or small intestine, the endogenous synthesis of arginine may not meet metabolic demands [20]. The availability of arginine for metabolic functions is determined by its transporters in the plasma and mitochondrial membranes. During critical illness, gastric emptying would be delayed and small intestinal and colonic motility reduced [11]. The rate-controlling enzymes in arginine synthesis and catabolism are argininosuccinate synthase, arginase isozymes, NOS, arginine decarboxylase [19]. Control is expressed according to cell type, age developmental stage, diet, and state of health and disease.

Four enzymes use arginine as a substrate: arginine decarboxylase, arginine:glycine amidinotransferase, arginase and its isoforms, and NOS [19-21]. The main products of the four enzymes are agmatine, guanidinoacetate and ornithine, ornithine and urea, and NO and citrulline. Arginine-derived guanidinoacetate is the immediate precursor for creatine, which maintains the energy metabolism of muscle, nerve and testis [112]. Creatine breaks down to creatinine at a constant rate and is cleared from the body by the kidneys.

In severe scurvy and in syndromes having pathological consequences, the correction of arginine deficiency could become a clinical priority. In critically ill patients, arginine maintains body homeostasis, muscle mass and function, and catabolic states [13,20]. It triggers the body to make protein, prevents the wasting, and is used in the management of wounds in the lower extremities [113]. Infectious complications become a serious problem [113,114]. Host invasion triggers an intense inflammatory response, which is then amplified by further pro-inflammatory cascades [111]. Severe cases are characterized by the rapid development of multiple organ failure resulting in mortality in excess of 40%. Dietary arginine supplementation helps to restore its plasma concentrations, boosts the immune system and helps to reduce neuropathy.

Clinical trials have used immune-enhancing enteral feeds that combine arginine with anti-inflammatory fatty acids, antioxidants, glutamine, and nucleotides. Glutamine has a potential beneficial metabolic effect in the critically ill [11], and in the treatment of infections in trauma patients [115]. Glutamine is the principal fuel of enterocytes and lymphocytes. While doubt has been raised as to the value of arginine supplementation [11], due to the lack of its exact role in the regulation of immune functions [111], the European Society of Parenteral and Enteral Nutrition has published guidelines recommending the routine use of arginine-containing diets in surgical patients [116]. An analysis of clinical studies using enteral formulas supplemented with arginine has suggested benefits upon outcomes from chronic and critical illness with little or no evidence of significant detrimental effects [117].

Today, vitamin and amino acid supplements have gained popularity as preventive or therapeutic agents. The excessive intake of high levels of a single amino acid or biofactor may be hazardous because of its potential for toxicity and control over genetic expressions. The effects of nutrients on gene expression are referred to as "nutrigenomics'. The study of the selective effects of arginine on gene expression is called 'argenomics'[19].

### Did nitric oxide aid in the recovery from scurvy?

While we may never know the answer to this question, the discovery of the significance of NO in the human body remains as one of the most important developments in recent medical history. In 1987, NO production from arginine was first identified in endothelial cells [118]. NO is a gas that transmits signals that are produced by one cell, penetrates through membranes, and regulates the function of another cell in humans and conifers (Figure 2). A recovery from scurvy involving NO occurs as a series of events over time. No model yet exists which integrates nutrition with vitamin C and NO in the Iroquois cure for scurvy.

In the severe winter at Stadaconna, the exposure of Cartier's crew to cold, poor diet, and insufficient vitamin C, would have benefited from the nutritional values of supplemental arginine, amino acids, and antioxidants. Boreal conifers lack nitrate because acid forest soils are poor in N and tissues contain little nitrate in the absence of fertilizers [7]. Nitrates and nitrites in the decoction would not have been available for their conversion to NO by the acidic gastric juice in the stomach. Readily available free arginine remains the main N source for NO which would kill almost all bacteria that were swallowed with food.

In clinical medicine and pharmacology, NO is a factor in treating intensive care patients [11-13,15,18,110,111]. In tissues, it regulates oxygen release from red blood cells. NO protects the heart, stimulates the brain, and regulates inflammation. It improves blood circulation for the provision of essential amino acids, antioxidants and vitamins to tissues. NO is important for olfactory function and memory. White blood cells use NO to kill infectious bacteria, fungi and parasites. It defends against tumors by inducing apoptosis. Dosage is critical since NO can be toxic at high concentrations.

Levels of vitamin C were depleted during the severely cold winter. The mitochondria, being the responsible subcellular organelles for the oxidation of dietary and remaining stored fat, would generate almost all of the energy needed by humans. The oxidation of arginine to NO and citrulline is exothermic and occurs in mitochondria. Shivering generates body heat from muscles. Mitochondria generate most of the ATP that provides the energy for metabolism to occur.

In tissues that consume ATP rapidly, such as brain, and skeletal and smooth muscle, phosphocreatine serves as an energy reservoir for the rapid regeneration of ATP [112]. Mitochondrial creatine kinase produces ATP from ADP by converting creatine phosphate to creatine in the mitochondrial intermembrane space. Creatine kinase is routinely determined in emergency patients because its elevated activity is an indication of damage to muscles, renal failure, and myocardial infarction. The oxidative reactions in mitochondria are also a major source of potentially damaging oxygen free radicals. Reactions with NO can remove these radicals, and stimulate the formation of new and larger mitochondria [119], which in turn would improve body bioenergetics.

Arginine is metabolized via mitochondria and in the cytosol via the urea cycle [10] (Figure 1). The cytosol makes up the cytoplasm of cells. It contains thousands of enzymes. Some act synergistically with vitamin C to provide a cure for scurvy and others aid in the recovery to health. NO, formed by the turnover of body proteins and supplemented by arginine from the decoction would reduce excessive stress, provide a targeted redistribution of energy resources to the vital organs, and activate a series of early and late-expressing genes as the body recovers. At the recovery stage, NO would participate in the maintenance of long-term health.

Arginine, amino acids and vitamin C are absorbed into circulation by the small intestine, and carried to collagen-rich cells throughout the body. Endothelial cells, which make up the capillaries and line blood vessels and the lymph ducts, use arginine to make NO. NO diffuses into the smooth muscle cells that surround the endothelial lining of blood vessels. This leads to muscle relaxation resulting in more blood flow to tissues, brain, lungs, kidneys, liver and other vital organs. Blood pressure is lowered. Blood flow and blood vessel diameter are improved, and the formation of blood clots would be reduced and repaired. The improvement of heart function would allow more oxygen, nutrients, vitamins, and amino acids to aid in the recovery from scurvy.

Improved circulation would relieve tension, sore muscles, the pain caused by swelling, and the accumulation of excess fluids in Cartier's crew and in Domagaia's legs. Wound healing would increase and mineral depletion would be reduced. NO is an important regulator in immune cells which fight infections and viruses. It aids in the killing of bacteria and engulfed pathogens found within the lysosomes of macrophages, which remove harmful cellular debris and dead cells. NO is a scavenger for cytotoxic free radicals that contribute to aging. NO, being involved in the transmission of messages between nerve cells connected with memory, sleeping and learning would support the wellness of sailors for their return to France.

Human NOS has three distinct isoforms having a multitude of organ-specific regulatory functions [18,19,21]. A constitutive form (nNOS) is found in neural tissue, and a second constitutive form (eNOS) is found in the vascular endothelium. Both are regulated by calcium and calmodulin. A third calcium-independent form (iNOS) occurs in a variety of cells after induction with inflammatory mediators and bacterial products. During inflammation, arginine transport increases [19]. The activation of iNOS or arginase, or both, reflects the type of inflammatory response in the development of specific diseases [111]. Sepsis, which occurred in Cartier's critically ill crew, probably involved a predominant role for arginine and iNOS [117]. Septic shock is a life threatening complication that can have a mortality rate of 50 to 80%. Trauma exhibits a preferential induction of arginase [111]. Arginase would reduce the arginine available as a substrate for NOS.

The formation of *S*-nitrosothiols provides a different mode of transporting NO, offers a buffering system that regulates the bioavailability of NO, and increases the range of NO action [120]. In plants and humans, glutamine serves as an antioxidant by enhancing the levels of glutathione resulting in the formation of *S*-nitrosoglutathione [11,22,121]. Some *S*-nitrosothiols (*S*-nitrosocysteine, *S*-nitrosohomocysteine) are taken up into cells via the amino acid transport system. *S*-Nitrosothiols are being considered as exogenous sources of NO for the treatment of NO-deficient pathological states. They are cleared to ameliorate nitrosative stress. They regulate innate immune and vascular function. *S*-nitrosylation may also contribute to the posttranslational modification of proteins involved in the regulation of NO action and in the recovery from scurvy. The nitrosylation of cysteine thiols is recognized as critical for the mechanism of NO function in health and disease.

Without clinical trials, it is not clear how quickly Cartier's crew would have recovered if given only vitamin C. It is evident that the decoction with vitamin C, arginine, the essential amino acids, antioxidants and other unidentified biofactors integrated with NO production in the body and aided in the recovery from scurvy.

### Guanidino compounds as NOS regulators and metabolic markers

The inappropriate release of NO is linked to the pathogenesis of a number of disease states. High levels of NO interact with molecular oxygen and superoxide radicals to produce damaging peroxynitrite that modifies proteins, lipids, and nucleic acids with harmful effects [122,123]. The need to regulate NO production has led to the development of modulators and inhibitors of NOS activity [18,21,122-125]. L-arginine analogues have the advantage of being closely related to the substrate itself and may be taken up by amino acid transporters. The guanidino compounds that interfere with the binding of arginine to NOS have been the most extensively researched as drugs [21].

In general, guanidino-substituted L-arginine molecules are effective but they show little selectivity for the NOS isoforms. *N*^*ω*^-Methylations of the guanidino moiety of L-arginine have yielded potent NOS inhibitors [21]. Their use in treating septic shock provided hemodynamic stabilization in a large number of patients. While *N*^*ω*^-methylated guanidino compounds have not yet been identified in conifers, they effectively inhibit NOS activity when fed to a variety of conifers [22,23,83,88,89]. L-arginine analogues with substitutions on their carbon chains, and lacking the α-amino group or α-carboxyl groups are inactive as inhibitors and as substrates for NOS. These include guanidinoacetic and γ-guanidobutyric acid.

Reviews of the physiological effects of natural and synthetic guanidino compounds in research and clinical practice are available in several books [126-128]. Among the known guanidino compounds in conifers, α-keto-δ-guanidinobutyric acid and γ-guanidinobutyric acid are found in nerve tissues [129] and in the urine of hyperarginemic patients [130]. Agmatine, a decarboxylation product of arginine, which occurs in conifers, has various pharmacological effects, but little is known about its metabolism and human physiology [19,20].

Guanidino compounds have been used as respiratory inhibitors, antibiotics, markers for metabolic disorders, and in studies of cardiovascular diseases, diabetes, and microbial activity [102,126-128]. Markers carry information about the sites and pathological cause. In uremia, guanidine, guanidinosuccinic acid and creatinine are greatly increased in biological fluids and tissues [131]. Guanidino compounds are implicated in hypertension, anaesthesia, hemorrhagic shock, seizure, renal dysfunction, and immersion stress [132]. γ-Guanidinobutyric acid reacts with hydroxyl radicals and induces epileptic discharges [127]. Its transamidination precursors are γ-aminobutyric acid and arginine [129].

Guanidine and the guanidino compounds have been used to treat botulism, viral diseases, diabetes, and paraneoplastic syndromes [132]. Evidence for mechanisms of action, dosing, pharmokinetics, cautions, interactions and clinical applications remains preliminary. Synthetic guanidino compounds are used in the treatment of diabetes [15]. Type II diabetes is one of the most serious health problems for Native Americans in the USA [133]. Pharmacogenetic differences, which diminish or enhance the predicted response, would indicate an inherited defect [107]. The surprisingly wide and natural diversity of chemically identified guanidino compounds in nature [134] may yet offer new biofactors and drugs in understanding ethnomedicine, in treating diseases, and for their contribution to regimens for human wellness.

## Conclusion

The history of the native indigenous peoples of eastern Canada has provided evidence of a culture strong enough to withstand the most difficult hardships. Their coniferous forests have had a pervasive influence on offering a source of food, fibre, protection, and medicinal products for human survival. In the absence of forensic evidence and apart from being a source of vitamin C, the formulary nature of the decoctions from Annedda to cure scurvy and hasten the recovery of the sailors is a story that may never be completely solved nor will it end here. We do not really know the true identity of Annedda. This tree, known today as arborvitae, has subsequently been represented by many candidate coniferous trees of life.

When food was short and the winter most severe, the candidate trees of life in eastern Canada provided a source of vitamins, arginine, proline, other conditionally and essential amino acids, antioxidants, and other biofactors, which aided in the recovery from of scurvy. The discovery of vitamins and amino acids played an important role in understanding human nutrition. For the discovery of the importance of arginine-derived NO in clinical nutrition we are indebted to the investigators who were honoured by the Nobel Prize in 1998.

After the transfer of the tree of life to France, old principles were now viewed in new ways. Jean Fernel (1497–1558), a foremost figure in the French Renaissance of science and medicine, broke away from the irrationality of occultism and magic that had dominated medieval natural philosophy and medicine [135]. His treatises on "Physiologia", first published in 1542, created debate in Europe for the next 100 years, and laid the "seeds of systems biology"[136]. To learn from history, we must transform it into a science.

Clinical nutrition is now faced with a vast amount of data from the ethnobotanical and ethnomedical literature. The detailed interdisciplinary and systematic understanding of the complex interactions among anatomy, histology, pathology, neurology, nutrition, and metabolism represents a modern-day challenge. The genetic loci of many controlling quantitative traits have been identified and are being compared in ill and healthy individuals [137-139]. Through genomics, argenomics, nutrigenomics, and other "omics" technologies, we expect to have a better understanding of how the control of complex traits would aid the recovery from sickness and diseases [19]. Integrated databases are being developed to describe the toxological, pharmaceutical, nutritional and environmental effects of diets and drugs on disease [140-143].

The history of medicine and clinical practice has involved a succession of blind alleys and detours, mountains of often uninterpretable observations, and a great leap forward as in the discovery of vitamin C as a cure for scurvy. This review takes us centuries back, and turns our attention to the combined values of arginine, NO, proline, other conditionally and essential amino acids, guanidino compounds, and antioxidants as added factors in the food and medicines of indigenous Canadian peoples.

## Competing interests

The author declares that they have no competing interests.
